# Enhanced Bioaccumulation and Toxicity of Arsenic in Marine Mussel *Perna viridis* in the Presence of CuO/Fe_3_O_4_ Nanoparticles

**DOI:** 10.3390/nano11102769

**Published:** 2021-10-19

**Authors:** Shuang Zhou, Wei Qian, Zigong Ning, Xiaoshan Zhu

**Affiliations:** 1 Shenzhen International Graduate School, Tsinghua University, Shenzhen 518055, China; mzhou0327@163.com (S.Z.); jokloy@126.com (W.Q.); 2 Shenzhen Honglue Research Institute of Innovation Management, Shenzhen 518119, China; 3 School of Environment, Tsinghua University, Beijing 100084, China; 4 School of Civil and Environmental Engineering, Harbin Institute of Technology, Shenzhen 518055, China; ningzigong@hit.edu.cn; 5 Southern Laboratory of Ocean Science and Engineering, Zhuhai 519000, China

**Keywords:** As, metal oxide NPs, detoxification, biotransformation, *Perna virids*

## Abstract

Leakage of metal oxide nanoparticles (MNPs) into marine environments is inevitable with the increasing use of MNPs. However, little is known about the effects of these lately emerged MNPs on the bioaccumulation and toxicity of pre-existing contaminants in marine biota. The current study therefore investigated the effects of two common MNPs, CuO nanoparticles (nCuO) and Fe_3_O_4_ nanoparticles (nFe_3_O_4_), on bioaccumulation and toxicity of arsenic (As) in green mussel *Perna viridis*. Newly introduced MNPs remarkably promoted the accumulation of As and disrupted the As distribution in mussels because of the strong adsorption of As onto MNPs. Moreover, MNPs enhanced the toxicity of As by disturbing osmoregulation in mussels, which could be supported by decreased activity of Na^+^-K^+^-ATPase and average weight loss of mussels after MNPs exposure. In addition, the enhanced toxicity of As in mussels might be due to that MNPs reduced the biotransformation efficiency of more toxic inorganic As to less toxic organic As, showing an inhibitory effect on As detoxifying process of mussels. This could be further demonstrated by the overproduction of reactive oxygen species (ROS), as implied by the rise in quantities of superoxide dismutase (SOD) and lipid peroxidation (LPO), and subsequently restraining the glutathione-S-transferases (GST) activity and glutathione (GSH) content in mussels. Taken together, this study elucidated that MNPs may elevate As bioaccumulation and limit As biotransformation in mussels, which would result in an enhanced ecotoxicity of As towards marine organisms.

## 1. Introduction

The commercial use of metal oxide nanoparticles (MNPs) has increased drastically over the decades in various industrial applications such as biochemical coatings, drug delivery, magnetic resonance imaging and catalysts, as well as in controlling environmental pollution [1,2,3,4,5,6]. Among these MNPs, CuO nanoparticles (nCuO) and Fe_3_O_4_ nanoparticles (nFe_3_O_4_) are importantly and commercially used nanomaterials, due to their unique properties such as large surface area, high surface reactivity and excellent affinity for heavy metals [7]. Nevertheless, the extensive use of nCuO and nFe_3_O_4_ in a variety of consumer products has resulted in their release into aquatic environment [8]. Unintentionally released nCuO and nFe_3_O_4_ may co-occur with pre-existing contaminants in aquatic environment, which would eventually alter the contaminants’ environmental behaviors, fate and toxicity to the ecosystem and even human health [9].

Arsenic (As) has been ubiquitously found in coastal regions, with a background concentration of up to dozens of μg/L due to anthropogenic processes (such as industrial, agricultural and mining effluents) [10,11]. As can be accumulated by marine organisms [12] and cause adverse biochemical and physiological effects such as immune disorders, reduced reproduction and growth, cell and tissue damage, and cell death [13]. However, there has been little information about the effect of newly emerged nCuO and nFe_3_O_4_ on the pre-existing As bioaccumulation and toxicity in marine organisms. Previous studies have focused on the ecotoxicity of target MNPs (including bacteria, protozoa, water flea, fish, bivalves and so on) rather than their effect on co-existing contaminants [14,15,16,17]. Interestingly, recent research found that TiO_2_ nanoparticles could inhibit biotransformation of inorganic As to organic As in the mussel *Perna viridis* [18], which confirmed that the MNPs could affect the toxicity of co-existing contaminants in aquatic organisms. However, it is still too little evidence to make comprehensive conclusions on the information about how the more recently introduced MNPs affect the behavior and toxicity of pre-existing contaminants in marine environments. More research is needed in this field to better assess the ecological risk of MNPs and As in marine environments.

Therefore, the purpose of this research is to investigate the effects of two typical MNPs (nCuO and nFe_3_O_4_) on the biotransformation and detoxification of As in the green mussel *Perna viridis* (*P. viridis*), following a series of long-term waterborne As exposures and As MNPs co-exposures. *P. viridis* is one of the most widely distributed bivalve species in marine environments. They are proficient at taking up suspended particles and accumulating contaminants under a wide range of environmental conditions [19,20] thus making them a good biomonitor and a key species for assessing the ecotoxicity of MNPs [21]. Here, we designed an aquaculture system to mimic the real exposure in marine environments where As exist chronically, and *P. viridis* already is acclimated to it. Then, the MNPs were considered as the new contaminant emerging in this system, hence providing opportunity to study their contributions to the ecotoxicity of As in *P. viridis.* We consequently analyzed As bioaccumulation and distribution, measured As speciation and As biotransformation-related biomarkers in order to facilitate the interpretation of the underlying mechanisms. We hypothesise that the lately introduced MNPs would enhance bioaccumulation and toxicity of As in marine mussel *P. viridis*. Overall, our findings would provide useful information for assaying the ecological risks of MNPs and As.

## 2. Materials and Methods

### 2.1. Chemicals and Nanoparticles Characterization

nCuO and nFe_3_O_4_ (<10 nm, purity ≥99.5%) stock suspensions were prepared in ultrapure water, sonicated (50 W, 40 kHz, KQ2200, Kun Shan Ultrasonic Instruments Co., Ltd, KunShan, China) for 30 min to reach a concentration of 1 mg/L, respectively. The MNPs were characterized prior to the toxicity tests. Specifically, the morphology of MNPs were analyzed at a dilution of 1 mg/L by transmission electron microscopy (TEM, Tecnai G2 Spirit, FEI, Hillsboro, OR, USA) and the particle sizes of MNPs during 24 h in seawater were determined at 20 °C by dynamic light scattering (DLS) with a zeta potential analyzer (Zeta-PALS, Brookhaven, Holtsville, NY, USA), more details on the characterization methods are described in Gomes et al. [3]. Na_2_HAsO_4_·7H_2_O was purchased from Sigma (Saint Louis, MO, USA), and the 1000 mg/L As(V) stock solution was made by dissolving it in artificial seawater.

### 2.2. Experimental Design

Green mussels *P. viridis* (n = 72, 6 ± 1.1 cm) were collected South of Guangdong Province (114°64′ E, 22°46′ N) and acclimated for seven days in artificial seawater at a constant temperature with aeration. After acclimation, half of the mussels were placed in 50 μg/L As(V) exposure media in a triplicate design, along with a control group kept in artificial seawater, for a period of 21 days. Water was completely changed every day with redosing after each change. Mussels were collected from control, 50 μg/L As(V) in the beginning of the experiment and after 1, 3, 7, 10, 14, and 21 days of exposure. After sampling, the mussels were washed, wet-weighed and stored at −80 °C for further use.

After 21 days of single As(V) exposure, co-exposure with MNPs were prepared by introducing 1 mg/L MNPs to control groups and 50 μg/L As(V) exposure groups (control groups: control + nCuO and control + nFe_3_O_4_, 50 μg/L As(V) exposure groups: As(V) + nCuO and As(V) + nFe_3_O_4_), for a period of 14 days, each co-exposure had three independent replicates. The concentration of MNPs selected was environmentally relevant. Three mussels were collected from control + nCuO, control + nFe_3_O_4_, As(V) + nCuO and As(V) + nFe_3_O_4_ in the beginning of the experiment and after 1, 3, 7, 10 and 14 days of exposure. After sampling, the mussels were washed, wet-weighed, dissected, and stored at −80 °C for further use. More details of the experiment design can be found in the Supplementary Materials.

### 2.3. Analysis of As in Mussels

The total As content in mussel was analyzed according to previously used methods [22] with minor modifications; microwave digestion was used to treat mussels in this study. The As concentration was measured by using an inductively coupled plasma mass spectrometer (Thermo Scientific^TM^ ICAP-Q, ICP-MS, Thermo Fisher Scientific, Waltham, MA, USA). The As species were extracted by using a two-step sequential extraction as described previously [23]. The concentration of As in mussels with different species (inorganic As(III) and As(V), organic monomethylarsonic acid (MMA), dimethylarsinic acid (DMA), and arsenobetaine (AsB)) was determined by using a Thermo Scientific IC5000 ion chromatography system combined with a Thermo Scientific^TM^ ICAP-Q, ICP-MS (IC-ICP-MS) [18].

### 2.4. As Bioconcentration Factor (BCF)

In the present study, we used BCF (which expresses the accumulation of a chemical substance directly from water through the gill apparatus and shells) to determine the accumulation of As in mussels based on the As concentration in the dry tissues of mussels. Specifically, BCF (L/kg, dw) was calculated as: BCF = As concentration in tissues (µg/g)/As concentration in culture water (mg/L).

### 2.5. As(V) Adsorption to MNPs in Seawater

Kinetic adsorption of As(V) onto MNPs were conducted in artificial seawater, using the same concentration as the exposure condition (50 μg/L As(V), 1 mg/L nCuO/nFe_3_O_4_). The details are provided in the Supplementary Materials.

### 2.6. Biomarkers Determination

Dried mussel tissues (50 mg) were homogenized with a 0.86% NaCl solution by using a tissue homogenizer. The supernatant was collected to assess the biomarkers after the homogenate of mussels’ tissues was centrifuged at 3500 rpm at 4 °C for 10 min. In this study, Na^+^-K^+^-ATPase (NKA), Superoxide dismutase (SOD) activity, lipid peroxidation (LPO) levels, reduced glutathione content (GSH) and glutathione-S-transferases (GST) in mussels’ tissues were chosen as the biomarkers, which were measured spectrophotometrically using the commercial kits (Nanjing Jiancheng Bioengineering Institute, Nanjing, China) on the basis of the manufacturers’ protocols. Specifically, the SOD activity was measured with a spectrophotometer (Milton Roy Spec20, Milton Roy Co., Rochester, NY, USA) at 550 nm, the LPO levels of different groups were detected using a thiobarbituric acid reactive substances (TBARS) assay, by measuring the amount of MDA-thiobarbituric acid (TBA) complex at 535 nm. In addition, the GSH content was estimated by the 5,5′ dithiobis-(2-nitrobenzoic acid) (DTNB)-glutathione reductase coupled assay at 420 nm, the GST was determined spectrophotometrically using commercially available GST activity kits based upon the GST-catalyzed reaction between glutathione (GSH) and the GST substrate 1-chloro-2,4-dinitrobenzene (CDNB) at 412 nm. The NKA activity was measured spectrophotometrically using NKA assay kit at 636 nm.

### 2.7. Statistical Analysis

SPSS version 19.0 (IBM, Armonk, NY, USA) was used to perform statistical analysis on the obtained data. As the data was normally distributed and the variances were homogenous, the differences within treated groups were evaluated by one-way analysis of variance (ANOVA) with Tukey’s posthoc tests. A probability level (*p*-value) of less than 0.05 was regarded as statistically significant.

## 3. Results and Discussion

### 3.1. MNPs Characterization

**Figure 1:**
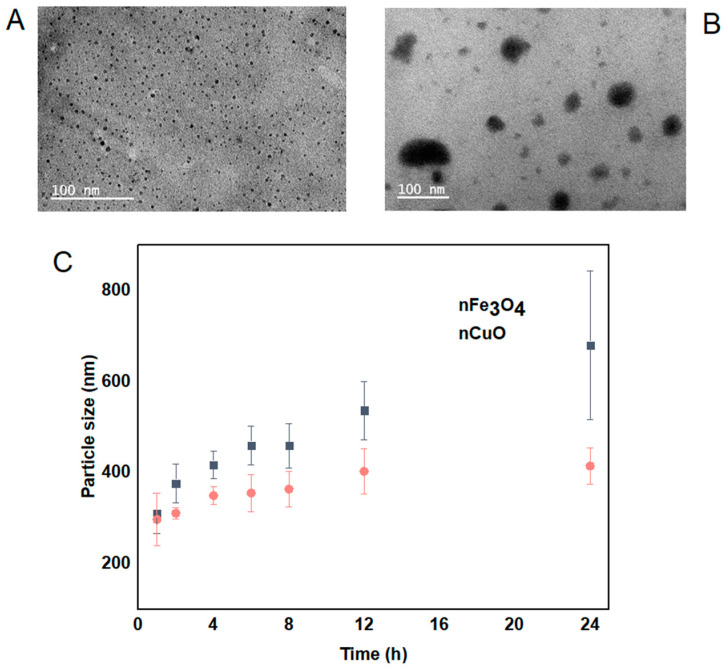
Transmission electron microscopic image of nCuO (**A**) and nFe_3_O_4_ (**B**). Particle size distribution (nm) during a 24 h time periods by dynamic light scattering for nFe_3_O_4_ and nCuO (**C**).

The morphology, size and distribution of nCuO and nFe_3_O_4_ were obtained by TEM analysis and DLS analysis. nCuO are spherical in shape with a mean size of 10 ± 3 nm. nFe_3_O_4_ are mainly spherical in shape and not strongly aggregated (Figure 1A,B). The size of both nCuO and nFe_3_O_4_ (<10 nm) reported by the manufacturer is broadly in agreement with the size obtained by TEM. In addition, we also determined the mean particle size by using DLS, both nCuO and nFe_3_O_4_ aggregated immediately when they were introduced to artificial seawater, and their sizes kept increasing during the 24 h exposure period (Figure 1C). Moreover, high polydispersity indexes were observed for nCuO (polydispersity index between 0.21 and 0.53) and nFe_3_O_4_ (polydispersity index between 0.22 and 0.56), suggesting that under the exposure conditions, both MNPs tendency to aggregate produces suspensions with the presence of both single particles and large aggregates, with size ranging from 10 to 414 nm for nCuO and 40 to 679 nm for nFe_3_O_4_. Several reports have shown the tendency of MNPs to form aggregates while in suspension by using the same particles [21,24,25].

### 3.2. Exposure to MNPs Increased As Bioaccumulation and Altered As Distribution in Mussels

**Figure 2:**
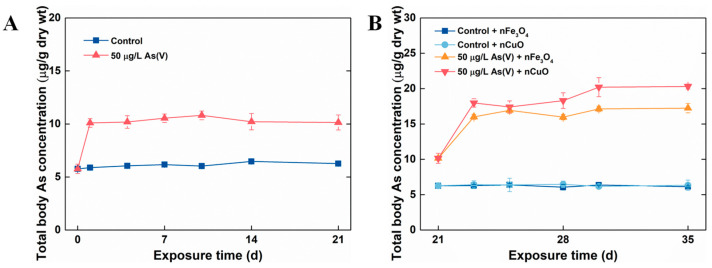
Dynamics of total body As concentrations in mussels without MNPs (**A**) and with MNPs (**B**). Data are mean ± SD (n = 4).

In the current study, *P. viridis* were firstly acclimated to the artificial seawater condition (as control mussels) and 50 μg/L As(V) exposure condition. Later, MNPs were introduced to the culture system in order to mimic the contaminated natural environment where As has pre-existed and MNPs were lately introduced as a new contaminant. The single As(V) exposure experiment showed that after 21 days exposure, no mortality was observed in both 50 μg/L As(V) exposure condition and control exposure condition, which confirmed that As could be detoxified in marine mussels [26]. Moreover, total body As concentrations in control mussels remained stable after 21 days exposure. However, the total body As concentration in mussels exposed to 50 μg/L As(V) after single As(V) exposure was significantly higher (10.14 ± 0.71 μg/g dw) in comparison to the ones in control mussels (6.26 ± 0.09 μg/g dw) (Figure 2A). These results validated that As can be accumulated and retained inside in mussels when mussels exposed to it through seawater, similar to the previous study in marine medaka (*Oryzias melastigma* and *Oryzias latipes*) [27,28].

After 21 days single As(V) exposure, co-exposure with MNPs were prepared by introducing 1 mg/L MNPs to control groups and 50 μg/L As(V) exposure groups (control groups: control + nCuO and control + nFe_3_O_4_, 50 μg/L As(V) exposure groups: As(V) + nCuO and As(V) + nFe_3_O_4_) for a period of 14 days. It was apparent that both nCuO and nFe_3_O_4_ did not change total body As concentrations in control + nCuO and control + nFe_3_O_4_ mussels. By contrast, As accumulation in As(V) + MNPs co-exposure mussels was increased (Figure 2B). Specifically, after co-exposure to As(V) + nFe_3_O_4_ for 14 days, the total body of As concentration in mussel increased from a concentration of 10.14 ± 0.71 μg/g dw to that of 17.23 ± 0.67 μg/g dw. Likewise, total body As concentration in mussel reached a new equilibrium (20.3 ± 0.36 μg/g dw) compared to a previous one (10.14 ± 0.71 μg/g dw) after co-exposure to As(V) + nCuO for 14 days (Figure 2B). Moreover, as the bioaccumulation potential of As by mussel can be measured by BCF value, the BCF for As(V) + nCuO and As(V) + nFe_3_O_4_ were calculated to be 406 L/kg dw and 344.6 L/kg dw at new equilibrium, respectively, which were remarkably higher than that (202.8 L/kg dw) in single As(V) exposure. These results implied that both nCuO and nFe_3_O_4_ could elevate As bioaccumulation in mussels.

The elevated As bioaccumulation in mussels could be due to the effectiveness of MNPs for adsorption of As [29,30]. Regarding metalloids such as As, previous studies identified the ‘Trojan horse effect’ consisting of MNPs capacity to adsorb co-existing pollutants and thus enabling for their uptake by organisms [31,32,33], which may increase their toxicity impacts. In the present study, we also investigated the adsorption of As onto nCuO/nFe_3_O_4_ in artificial seawater. There was a rapid uptake of As(V) in the first 30 min, and then it reached equilibrium. The adsorption equilibrium of As(V) on nCuO and nFe_3_O_4_ were 86.37% and 80.21%, respectively (Figure S1). In addition, as introduced in the first section, *P. viridis* are proficient at taking up suspended particles and accumulating contaminants, thus, mussels might increase the total As concentrations in their body effectively by filter water and suspended particles containing As.

**Figure 3:**
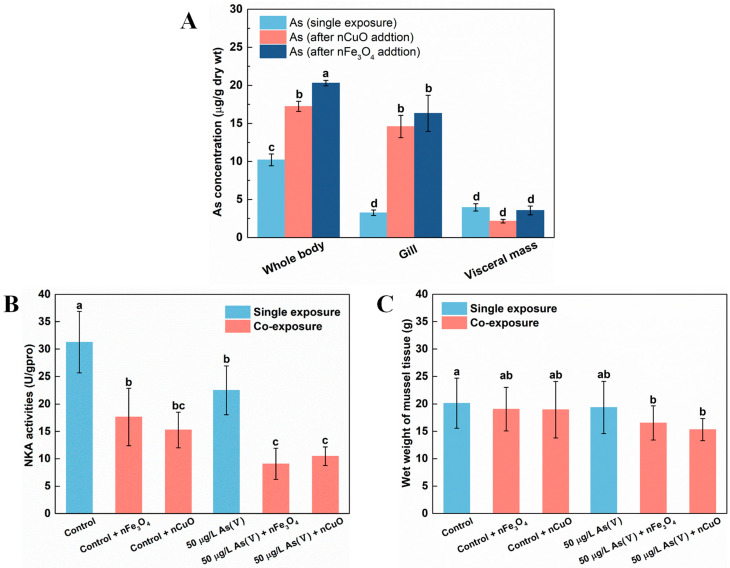
As Distribution before and after exposed to nFe_3_O_4_ and nCuO in mussels (**A**). Na^+^-K^+^-ATPase (NKA) activities in mussels exposed to different treatments at the end of the exposure (**B**). Body weight of mussels at the end of single As(V) exposure and co-exposure (**C**). Values are the mean ± SD (n = 6). Significant differences (*p* < 0.05) among exposure conditions were represented with different letters. Control: Mussels exposed to artificial seawater for 21 days (single exposure). Control + nCuO: Mussels exposed to 1 mg/L nCuO for 2 weeks after control exposure (co-exposure). Control + nFe_3_O_4_: Mussels exposed to 1 mg/L nFe_3_O_4_ for 2 weeks after control exposure (co-exposure). 50 μg/L As(V) only: Mussels were cultured in 50 μg/L As(V) solution for 21 days (single exposure). 50 μg/L As(V) + nCuO: Mussels exposed to 50 μg/L As(V) + nCuO for 2 weeks after 50 μg/L As(V) exposure (co-exposure). 50 μg/L As(V) + nFe_3_O_4_: Mussels exposed to 50 μg/L As(V) + nFe_3_O_4_ for 2 weeks after 50 μg/L As(V) exposure (co-exposure).

In addition, as previous researchers have pointed out the excellent affinity of nCuO and nFe_3_O_4_ towards As [7], it is likely that the distribution of As among mussels’ tissues would be disrupted due to this unique property of MNPs. Thus, we analyzed As distribution in both single As(V) exposure and co-exposure mussels. The results showed that As was mainly retained in visceral mass under single As(V) exposure (Figure 3A). However, the distribution of As in mussels’ tissues altered remarkably where gill was the main tissue for As retaining rather than visceral mass under co-exposure with As+MNPs. Furthermore, the visceral mass retained ever lower As concentrations (although not significantly) after co-exposure with MNPs compared to that in single As(V) exposure mussels (Figure 3A). This indicated that although As ingestion by mussels was elevated after co-exposure to either one of nCuO and nFe_3_O_4_, gill was the main tissue responsible for As retaining instead of visceral mass, resulting in higher stress levels in gill tissue [34,35].

### 3.3. Exposure to MNPs Enhanced the Toxicity of As in Mussels

It is possible that the toxicity of As for mussels might be enhanced since the As bioaccumulation and distribution in mussels significantly changed after exposure to MNPs. As mentioned above, the distribution of As in mussels’ tissues altered remarkably under co-exposure with As + MNPs, where gill was the main tissue responsible for As retaining instead of visceral mass. Gill, as a unique organ for mussel osmoregulation, might be damaged by the elevated As concentrations [36]. To test our hypothesis, we assessed the osmoregulation capacity of gill by estimating the activities of Na^+^-K^+^-ATPase (NKA), since NKA is important not only for osmoregulation, but also for providing a driving force for many transporting systems in marine organisms [36]. Indeed, NKA activities decreased after exposure to nCuO/nFe_3_O_4_ (Figure 3B), which confirmed the osmoregulation disorder in mussels after MNPs exposure. Specifically, compared with the control exposure and As(V) single exposure, the NKA activity after exposure to nCuO/nFe_3_O_4_ was decreased by 51.1%/43.8% and by 53.3%/59.6%, respectively (Figure 3B). The energy metabolism of mussels was inhibited by the decreasing of osmoregulation capacity of mussels due to the decrease in NKA activity, causing a potentially physiological response, which resulted in affecting the growth of mussels. Although no mortality was observed in both single As(V) exposure condition and co-exposure condition, the average body weight of mussels after As(V) + MNPs exposure were significantly lower than that of the mussels in single As(V) exposure (Figure 3C), which confirmed the increased toxicity toward mussels after MNPs exposure.

Previous studies have reviewed that As could be detoxified in aquatic organisms through a series of detoxification strategies. One of the main strategies is As biotransformation. On the one hand, marine organisms could reduce less toxic As(V) to more toxic As(III) and subsequently excrete it, since As(III) is more easy to excrete compared to As(V) [37]. On the other hand, marine organisms can firstly reduce As(V) to As(III), afterwards, As methylation process occurred where As(III) was methylated to organic As species such as MMA, DMA and AsB [26,38]. Thus, the enhanced toxicity of As in mussels could also be attributed to the disrupting of As biotransformation in mussels after addition of MNPs. To test our hypothesis, we then analyzed the contents of both organic As species (i.e., MMA, DMA and AsB) and inorganic As species (i.e., As(III) and As(V)) in mussels after both single As(V) exposure and co-exposure.

**Figure 4:**
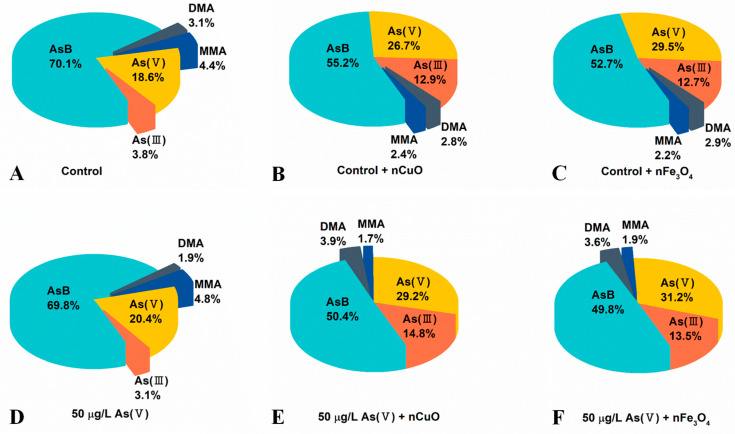
Content of As speciation in mussels after different exposure (organic monomethylarsonic acid (MMA), dimethylarsinic acid (DMA), and arsenobetaine (AsB)). (**A**) Control: Mussels exposed to artificial seawater for 21 days. (**B**) Control + nCuO: Mussels exposed to 1 mg/L nCuO for 2 weeks after control exposure. (**C**) Control + nFe_3_O_4_: Mussels exposed to 1 mg/L nFe_3_O_4_ for 2 weeks after control exposure. (**D**) 50 μg/L As(V) only: Mussels were cultured in 50 μg/L As(V) solution for 21 days. (**E**) 50 μg/L As(V) + nCuO: Mussels exposed to 50 μg/L As(V) + nCuO for 2 weeks after 50 μg/L As(V) exposure. (**F**) 50 μg/L As(V) + nFe_3_O_4_: Mussels exposed to 50 μg/L As(V) + nFe_3_O_4_ for 2 weeks after 50 μg/L As(V) exposure.

As shown in Figure 4 and Table S1, the concentrations of organic As species and inorganic As species increased in mussels after co-exposure to As(V) + nCuO and As(V) + nFe_3_O_4_, which confirmed an elevated As bioaccumulation in mussels due to the lately introduced nCuO and nFe_3_O_4_. Surprisingly, after co-exposure to As(V) + nCuO and As(V) + nFe_3_O_4_, the percentage of inorganic As species in mussels increased from 23.5% to 44.0% and 44.7%, respectively (Figure 4D–F, Table S1). As a result, the percentage of organic As in mussels after co-exposure to As(V) + nCuO and As(V) + nFe_3_O_4_ decreased from 76.5% to 56.0% and 55.3%, respectively (Figure 4D–F, Table S1). Particularly, inorganic As to organic As ratios in mussels after co-exposure to As(V) + nCuO and As(V) + nFe_3_O_4_ were 0.81 and 0.79, respectively, which were significantly higher than that (0.30) before the introduction of nCuO and nFe_3_O_4_ (Table S1). These results therefore implied that nCuO and nFe_3_O_4_ may restrict As biotransformation by limiting the transformation of inorganic As to organic As.

More importantly, As(V) to As(III) ratio was 6.54 in mussels before introduction of nCuO/nFe_3_O_4_, it dramatically decreased to 1.97 and 2.31 in mussels after As(V) + nCuO and As(V) + nFe_3_O_4_ exposure, which indicated a more efficient As(III) bioaccumulation than that of As(V) bioaccumulation in mussels after introduction of nCuO/nFe_3_O_4_. On the other hand, the proportion of MMA after As(V) + nCuO and As(V) + nFe_3_O_4_ exposure were less than that of MMA in mussels before As(V) + nCuO and As(V) + nFe_3_O_4_ exposure. It has been reported that organic MMA is the main product in the biotransformation of inorganic As(III) during the As methylation process, which is the crucial stage for As detoxification in mussels [39], the higher efficient As(III) bioaccumulation and less MMA proportion in mussels both implied that As methylation process (i.e., transformation of inorganic As forms to organic As forms) was inhibited by MNPs. In addition, As methylation process inhibited by MNPs was further demonstrated by the experiment of mussel exposure to nCuO/nFe_3_O_4_ only (Figure 4B,C and Table S1). Without another introduced As (i.e., no other As sources), there is a strong possibility that the variation of different pre-existing As species proportion in mussels was due to the intervention of nCuO/nFe_3_O_4_ on As biotransformation. As a whole, the results showed that the average body weight loss of mussels attributed to the enhanced toxicity of As, because the presence of MNPs led to the increasing inorganic As contents and decreasing organic As contents in mussels.

**Figure 5:**
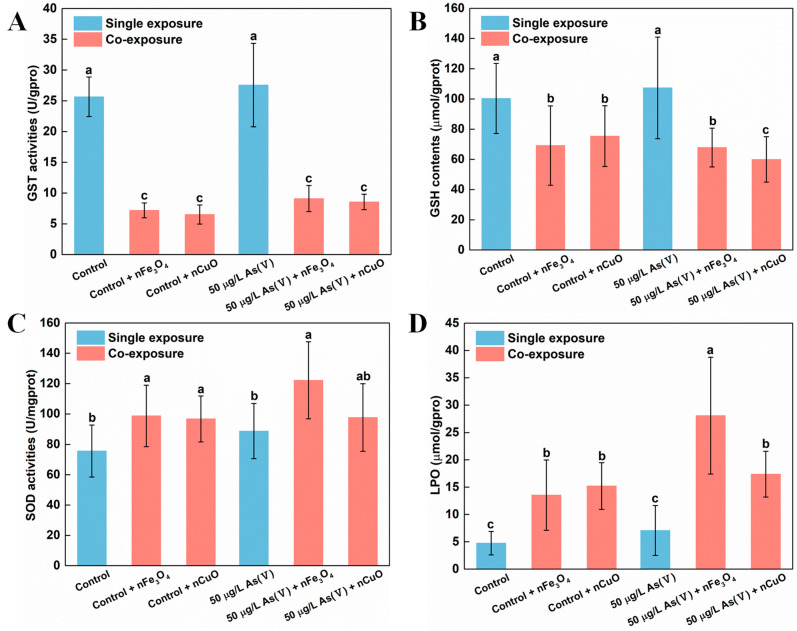
Biomarker values in mussels after different exposure. (**A**) GST activities, (**B**) GSH contents, (**C**) SOD activities and (**D**) LPO levels in mussel exposed to different treatments at the end of the exposure (mean ± SD, n = 6). Significant differences (*p* < 0.05) among exposure conditions were represented with different letters. Control: Mussels exposed to artificial seawater for 21 days (single exposure). Control + nCuO: Mussels exposed to 1 mg/L nCuO for 2 weeks after control exposure (co-exposure). Control + nFe_3_O_4_: Mussels exposed to 1 mg/L nFe_3_O_4_ for 2 weeks after control exposure (co-exposure). 50 μg/L As(V) only: Mussels were cultured in 50 μg/L As(V) solution for 21 days (single exposure). 50 μg/L As(V) + nCuO: Mussels exposed to 50 μg/L As(V) + nCuO for 2 weeks after 50 μg/L As(V) exposure (co-exposure). 50 μg/L As(V) + nFe_3_O_4_: Mussels exposed to 50 μg/L As(V) + nFe_3_O_4_ for 2 weeks after 50 μg/L As(V) exposure (co-exposure).

Existing research recognized that GST was a biotransformation rate limiting enzyme in mussels which have significant effect on As biotransformation and detoxification processes [22]. Moreover, GSH might also bind to As(V) which was a common and important mechanism during As metabolism [40]. For that reason, we measured GST activity and GSH content in mussels to elucidate their functions during As biotransformation and detoxification processes [22,41,42,43]. Indeed, our previous study about the nTiO_2_ effect on biotransformation of As in mussels showed that the biotransformation of As was limited because of the down-regulated GST and GSH content in mussels [18]. In the current study, the decreasing of GST activity and GSH content in mussels were similar to those described in our previous study [18]. After exposing to As(V) + nCuO and As(V) + nFe_3_O_4_, compared with single 50 μg/L As(V) exposure, GST activity greatly decreased by 84.8% and 67.0% in 50 μg/L As(V) co-exposure, respectively. GST activity also decreased by 74.6% and 71.9% in control + nCuO and control + nFe_3_O_4_ mussels, respectively, compared with single control exposure (Figure 5A). Likewise, there was a similar reduction in the GSH contents, in As(V) + nCuO and As(V) + nFe_3_O_4_ exposure, which was lowered by 43.9% and 36.4% than in 50 μg/L As(V) exposure, respectively. It also decreased by 24.6% and 31.0% in control + nCuO and control + nFe_3_O_4_ mussels, compared with the control exposure, respectively (Figure 5B). All these results indicate that MNPs can mediate biotransformation of inorganic As to organic As in mussels by decreasing As metabolism enzymes such as GST and GSH, thereby enhancing toxicity of As towards mussels.

Another probable explanation for the inhibitory effects of MNPs on As biotransformation and detoxification in mussels might be that the specific surface characteristics as well as chemical properties enable NPs the capacity to generate ROS by interaction with subcellular structures [3]. In the case of MNPs, the physical contact between mussels and particles may cause overproduction of ROS, leading to an increase in activity of antioxidant enzymes such as SOD and LPO [44,45,46]. In the present study, the generation of ROS was confirmed after exposure to nCuO/nFe_3_O_4_. Both SOD activities and LPO levels increased in mussels exposed to nCuO/nFe_3_O_4_ compared with the control groups without nCuO/nFe_3_O_4_ exposure (Figure 5C,D). Accordingly, nCuO and nFe_3_O_4_ might inhibit the As biotransformation in mussels through overproduction of ROS. These inhibitory effects imply an overproduction of ROS that could have led to the degeneration of As metabolism related enzymes such as GST and GSH [47], which could further support the decreasing of GST activity and GSH content in mussels and average weight loss of mussels after MNPs exposure. According to these results, it can be inferred that mussels could be subjected to a more toxic As environment after MNPs exposure.

## 4. Conclusions

The MNPs such as nCuO and nFe_3_O_4_ are increasingly applied in a variety of areas, and it is possible that they will end up in the environment in significant quantities, which makes it important to identify its effect on surrounding biota and environment. This study highlighted the importance of MNPs on biotransformation and toxicity of arsenic in green mussel *Perna viridis*. Exposure to MNPs elevated the bioaccumulation of As(V) and altered the As distribution in mussels, these alterations could be attributed to the adsorption of As on MNPs. What is more, newly introduced MNPs disturbed the osmoregulation system and enhanced the toxicity of As in mussels, which could be supported by decreased activities of Na^+^-K^+^-ATPase and average weight-loss of mussels after MNPs exposure. The present MNPs in mussels increase the content of inorganic As and motivate the ROS generation. The overproduction of ROS (SOD and LPO) restrains the activities of As metabolism enzymes (GST and GSH) in mussels, and then reduces As methylation and detoxification, subsequently, resulting in an increase in the toxicity of As to the mussels. The current work validates that MNPs enhance the bioaccumulation and toxicity of As in marine biota, resulting in an enhanced ecotoxicity of As towards marine ecosystems, and which improve our understanding about the ecological risks of MNPs and As.

## Supplementary Materials

The following are available online at https://www.mdpi.com/article/10.3390/nano11102769/s1. Figure S1: As(V) adsorption onto 1 mg/L nCuO and nFe_3_O_4_, Table S1: As species concentrations (µg/g) and proportion (%). Supplementary data to this research article can be found in Supplementary Materials.
