# Supplementary material for: Enhanced Bioaccumulation and Toxicity of Arsenic in Marine Mussel Perna viridis in the Presence of CuO/Fe3O4 Nanoparticles
Source: Nanomaterials (Basel). 2021 Oct 19;11(10):2769. doi: 10.3390/nano11102769 (PMC8538411; doi:10.3390/nano11102769)
Supplement: Supplementary file 1 [file nanomaterials-11-02769-s001.zip › nanomaterials-1379409-supplementary.pdf]

nanomaterials

MDPI

Supplementary Materials

Enhanced Bioaccumulation and Toxicity of Arsenic in Marine Mussel *Perna viridis* in the Presence of CuO/Fe_3O_4 Nanoparticles

Shuang Zhou [1,2], Wei Qian [1,3], Zigong Ning [4] and Xiaoshan Zhu [1,5,*]

[1] Shenzhen International Graduate School, Tsinghua University, Shenzhen 518055, China; mzhou0327@163.com (S.Z.); jokloy@126.com (W.Q.)
[2] Shenzhen Honglue Research Institute of Innovation Management, Shenzhen 518119, China
[3] School of Environment, Tsinghua University, Beijing 100084, China
[4] School of Civil and Environmental Engineering, Harbin Institute of Technology, Shenzhen 518055, China; ningzigong@hit.edu.cn
[5] Southern Laboratory of Ocean Science and Engineering, Zhuhai 519000, China
* Correspondence: zhu.xiaoshan@sz.tsinghua.edu.cn

Preparation of Artificial Seawater

Artificial seawater was prepared by dissolving commercial sea salts (Tianjin Cnsic Marine Biotechnology Company Limited, Tianjin, China) in ultrapure water (Millipore, Billerica, MA, USA), with a salinity = 30 ± 2 ‰, pH = 8.0 ± 0.2, total As < 0.5 μg/L, and dissolved oxygen concentration ≥ 7.0 mg/L. Testing solutions were prepared immediately before use by diluting the stock solution with artificial seawater.

Experimental Design

In the present study, the mussels were randomly allocated into each experimental tank (4 L of exposure solution) at a density of 12 per tank with continuous air supply to maintain dissolved oxygen near saturation, all exposure solutions were prepared immediately before use by diluting the stock solution with artificial seawater, and mussels were exposed to As(V) condition without any potential influences of food on As uptake. Specifically, the mussels were exposed to As(V) for 22 h and fed with 1 × 105 cells/mL Phaeodactylum tricornutum for 2 h in clean artificial seawater to avoid any potential influences of food on As uptake every day. Temperature (16 ± 1.1°C), salinity (30.3 ± 0.3) and pH (8.0 ± 0.3) were measured daily. The exposure water was completely renewed with artificial seawater containing As(V) every day after feeding. The mussels were collected prior to the feedings and were then shucked and rinsed with ice-cold 0.86% (w/v) NaCl solution, wet-weighed, and stored at −80°C for determination of body total As concentration, As speciation, and associated biomarkers. All treatments were performed in triplicate.

As(V) Adsorption to MNPs in Artificial Seawater

The adsorption kinetics of As(V) to either of nCuO and nFe3O4 were determined in artificial seawater (16℃, salinity = 30 ± 2‰, pH = 8.0 ± 0.2, total As < 0.5 μg/L, and dissolved oxygen concentration ≥ 7.0 mg/L). At first, either nCuO or nFe3O4 were mixed with As(V) in glass beakers, with the final concentration of 1 mg/L and 50 μg/L, respectively. Then, the mixtures were sampled at 30, 60, 120, 180 min and 240 min, and centrifuged at 12,000 rpm for 10 min. The supernatant was filtered through a 0.22-μm Teflon membrane and the As(V) concentration was measured by ICP-MS. Each assay was performed in triplicate. The results have been summarized in Figure S1. To account for the adsorption of As(V) to the glass walls of the beakers and the surfaces of the Teflon membrane, assays with 50 μg/L As(V) alone was also conducted as control. The results

indicated that cumulative adsorption of As(V) to the glass walls and the membrane surfaces was less than 5.0 % of the total amount, which was considered acceptable for this analysis.

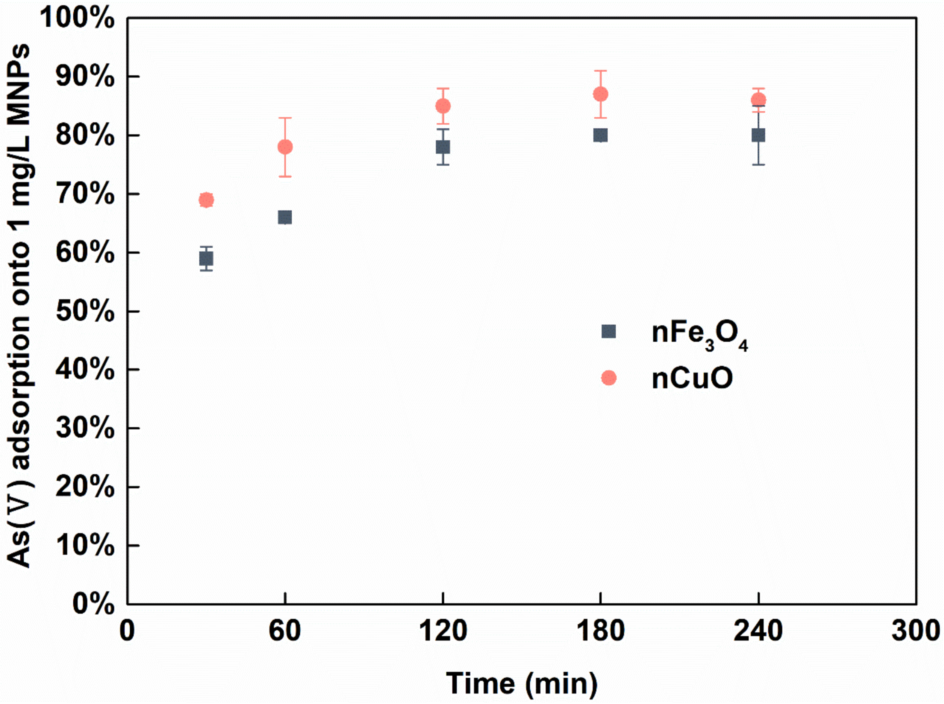

Figure S1. As(V) adsorption onto 1 mg/L nCuO and nFe_3O_4. Values are the mean ± SD (n = 3).

Table S1. As species concentrations (μg/g) and proportion (%). Values are means ± SD (n = 6). Different letters indicate statistically significant difference between different exposure time ($p < 0.05$).

Exposure time	AsB	DMA	MMA	Organic As	As(V)	As(III)	Inorganic As	Inorganic As/ Organic As
Control-0d	**2.65±0.21[a]** (82.08±6.85[a])	**0.05±0.02[c]** (1.42±0.13[c])	**0.04±0.01[c]** (1.28±0.34[c])	**2.74±0.19[b]** (84.8±9.27[a])	**0.41±0.03[e]** (12.85±0.48[d])	**0.08±0.02[c]** (2.37±0.36[c])	**0.49±0.02[e]** (15.2±0.11[e])	0.17
Control-7d	**2.44±0.36[a]** (76.13±11.32[a])	**0.07±0.03[ab]** (2.25±2.05[ab])	**0.09±0.01[b]** (2.68±0.25[b])	**2.60±0.43[ab]** (81.1±12.54[b])	**0.54±0.06[c]** (16.98±2.68[c])	**0.06±0.01[c]** (1.95±0.14[c])	**0.61±0.03[d]** (18.9±0.21[d])	0.23
Control-14d	**2.67±0.32[a]** (71.25±9.32[a])	**0.10±0.01[ab]** (2.79±0.35[ab])	**0.13±0.02[b]** (3.35±0.78[ab])	**2.90±0.32[ab]** (77.4±8.65[a])	**0.78±0.04[c]** (20.85±1.12[b])	**0.07±0.03[c]** (1.77±1.32[c])	**0.85±0.10[c]** (22.6±0.08[c])	0.29
Control-21d	**2.28±0.19[a]** (70.12±1.45[a])	**0.11±0.01[a]** (3.10±0.43[a])	**0.15±0.03[a]** (4.43±0.67[a])	**2.55±0.08[a]** (77.6±1.85[a])	**0.65±0.01[b]** (18.62±0.44[b])	**0.13±0.05[c]** (3.81±1.25[c])	**0.76±0.06[c]** (22.4±0.05[c])	0.31
nCuO -7d	**2.18±0.12[b]** (57.20±4.13[b])	**0.07±0.01[bc]** (1.91±0.23[bc])	**0.11±0.01[b]** (3.09±0.83[b])	**2.36±0.11[c]** (62.2±5.14[c])	**1.00±0.05[b]** (27.64±1.99[a])	**0.37±0.02[b]** (10.16±0.94[b])	**1.37±0.12[b]** (37.8±0.10[b])	0.58
nCuO -14d	**1.90±0.14[b]** (55.21±5.35[b])	**0.09±0.01[c]** (2.83±0.09[bc])	**0.08±0.02[b]** (2.41±0.74[b])	**2.09±0.16[b]** (60.4±5.99[c])	**0.84±0.07[b]** (26.69±2.32[a])	**0.41±0.01[b]** (12.91±1.32[ab])	**1.25±0.12[b]** (39.6±0.04[ab])	0.60
nFe_3O_4 -7d	**2.08±0.18[b]** (58.31±4.56[b])	**0.05±0.02[c]** (1.46±0.14[bc])	**0.10±0.02[b]** (2.94±0.88[b])	**2.23±0.10[c]** (62.7±5.10[b])	**1.03±0.06[b]** (30.42±2.02[a])	**0.23±0.04[b]** (6.88±0.56[b])	**1.26±0.10[b]** (37.3±0.03[b])	0.57
nFe_3O_4 -14d	**1.80±0.11[c]** (52.71±6.32[b])	**0.10±0.03[c]** (2.92±0.12[bc])	**0.08±0.03[b]** (2.19±0.69[b])	**1.98±0.12[c]** (57.8±8.25[b])	**1.01±0.11[a]** (29.53±1.98[a])	**0.44±0.02[a]** (12.71±1.16[b])	**1.47±0.03[a]** (42.2±0.02[b])	0.74
As(V)-0d	**2.65±0.21[a]** (82.08±6.85[a])	**0.05±0.02[c]** (1.42±0.13[c])	**0.04±0.01[c]** (1.28±0.34[c])	**2.74±0.19[b]** (84.8±9.27[a])	**0.41±0.03[e]** (12.85±0.48[d])	**0.08±0.02[c]** (2.37±0.36[c])	**0.49±0.02[e]** (15.2±0.11[e])	0.17

As(V)-7d	**3.58±0.24^{b}**<br>(65.26±3.26^{b})	**0.09±0.01^{c}**<br>(1.64±0.38^{b})	**0.20±0.01^{b}**<br>(3.64±0.55^{b})	**3.87±0.12^{c}**<br>(70.5±3.48^{b})	**1.45±0.04^{c}**<br>(26.47±0.15^{c})	**0.16±0.02^{c}**<br>(2.99±0.23^{c})	**1.62±0.02^{c}**<br>(29.5±0.34^{c})	0.42
As(V)-14d	**4.18±0.13^{b}**<br>(70.64±4.52^{b})	**0.07±0.03^{c}**<br>(3.15±0.47^{b})	**0.36±0.03^{a}**<br>(2.35±0.65^{a})	**4.63±0.20^{b}**<br>(78.1±4.97^{ab})	**1.23±0.03^{d}**<br>(20.84±0.11^{c})	**0.06±0.02^{c}**<br>(1.02±0.39^{c})	**1.29±0.04^{d}**<br>(21.9±0.14^{d})	0.28
As(V)-21d	**3.68±0.08^{b}**<br>(69.83±5.97^{b})	**0.10±0.02^{c}**<br>(1.89±0.21^{b})	**0.25±0.02^{a}**<br>(4.83±0.35^{a})	**4.03±0.11^{b}**<br>(76.5±5.12^{ab})	**1.08±0.02^{d}**<br>(20.41±3.98^{c})	**0.16±0.03^{c}**<br>(3.12±1.35^{c})	**1.24±0.11^{d}**<br>(23.5±2.15^{d})	0.30
As(V + nCuO -7d	**5.44±0.11^{c}**<br>(58.36±2.92^{c})	**0.42±0.03^{b}**<br>(3.55±0.23^{a})	**0.18±0.01^{b}**<br>(2.15±0.32^{c})	**6.05±0.20^{a}**<br>(64.1±4.23^{b})	**3.15±0.04^{b}**<br>(27.65±1.94^{b})	**1.60±0.01^{b}**<br>(8.29±1.04^{b})	**4.75±0.15^{b}**<br>(35.9±2.05^{b})	0.79
As(V) + nCuO -14d	**5.63±0.09^{c}**<br>(50.42±4.65^{d})	**0.44±0.01^{a}**<br>(3.93±1.65^{a})	**0.19±0.01^{b}**<br>(1.72±0.43^{c})	**6.25±0.46^{a}**<br>(56.0±5.58^{c})	**3.26±0.05^{a}**<br>(29.23±0.73^{a})	**1.65±0.02^{a}**<br>(14.81±2.56^{a})	**4.91±0.21^{a}**<br>(44.0±3.04^{a})	0.79
As(V) + nFe_3O_4-7d	**5.12±0.08^{a}**<br>(54.35±4.36^{d})	**0.30±0.01^{a}**<br>(3.15±0.19^{a})	**0.22±0.02^{b}**<br>(2.35±0.37^{c})	**5.64±0.37^{a}**<br>(59.8±7.02^{c})	**2.98±0.02^{a}**<br>(31.64±0.43^{a})	**0.80±0.02^{a}**<br>(8.51±3.27^{a})	**3.78±0.25^{a}**<br>(40.2±1.98^{a})	0.67
As(V) + nFe_3O_4-14d	**4.72±0.01^{a}**<br>(49.83±3.97^{d})	**0.34±0.02^{a}**<br>(3.62±1.07^{a})	**0.18±0.01^{b}**<br>(1.89±0.46^{c})	**5.24±0.38^{a}**<br>(55.3±5.69^{c})	**2.96±0.03^{a}**<br>(31.22±0.66^{a})	**1.28±0.01^{a}**<br>(13.51±2.94^{a})	**4.24±0.32^{a}**<br>(44.7±4.57^{a})	0.81
